# Protein for Community-Dwelling Older People: Aspects That Influence the Perception of Commercially Available Protein Drinks

**DOI:** 10.3389/fnut.2020.00100

**Published:** 2020-07-28

**Authors:** Lyn Lampmann, Anne Hannink, Eva Kiesswetter, Agnes Emberger-Klein, Dorothee Volkert, Klaus Menrad

**Affiliations:** ^1^Chair of Marketing and Management of Biogenic Resources, Weihenstephan-Triesdorf University of Applied Sciences, Technical University of Munich Straubing for Biotechnology and Sustainability, Straubing, Germany; ^2^Institute for Biomedicine of Aging, Friedrich-Alexander University of Erlangen–Nuremberg, Nuremberg, Germany

**Keywords:** perception, protein, older people, focus groups, enable cluster

## Abstract

In an aging population, support for independent living is increasingly critical for older generations. Currently, sarcopenia is a major cause of frailty, which increases the risk of decreased mobility, falls, morbidity, and mortality and leads to dependence on third parties. Sarcopenia is preventable by consumption of adequate protein. However, many older people do not meet the recommended daily allowance of protein, thereby supporting dependence rather than independent living. Current literature indicates that a protein drink could be an appropriate product for older peoples' protein consumption. We were interested in autonomous persons whose nutritional decisions were still self-determined and thus could preventively influence their personal health. This study evaluated three commercially available protein drinks in three focus groups (*n* = 25) to gain insight into which aspects influence the perception of commercial protein drinks on community-dwelling older people (age, 76.8 ± 4.9). Findings from the focus groups revealed only aspects, which influenced the perception of commercial protein drinks negatively. Most importantly, the drinks did not comply with relevant aspects when buying (healthy) foods, which where naturalness, freshness, locally grown ingredients, and trust. Furthermore, the target group did not see a need for additional protein consumption. Thus, we identify important aspects to be considered for the development of a target-group-specific protein drink as well as more suitable communication to prevent distrust in order to support independent living for community-dwelling older people.

## Introduction

In an aging population, maintaining physical function and mobility as well as enabling independent living is highly important for older people aged 65 years or more (1, 2). An essential factor as to why older people cannot live independently is the age-related loss of muscle mass, muscle strength, and function, called sarcopenia. Sarcopenia is a major cause of frailty in the elderly and increases the risk of mobility limitations, falls, morbidity, and mortality (3–5). One identified risk factor of sarcopenia is low protein intake (6). Large prospective cohort studies provided evidence that a high dietary protein intake is associated with a reduction in the decline of muscle mass, strength, and function in older people (6–8). However, many older people do not meet the recommended daily intake of 0.8 g protein/kg of body weight per day (6, 9–11). Additionally, there is growing evidence that older people even have higher protein requirements of 1.0–1.2 g/kg of body weight per day in order to maintain muscle mass and function (1, 12, 13).

Within this study, we were especially interested in autonomous persons, whose decisions were still self-determined. Therefore, we argue in favor of a product that has preventive characteristics and can be readily implemented in the diet of older people before they suffer from frailty to support their independent lifestyle as long as possible.

Currently, no adequate strategy seems to exist to guarantee an appropriate amount of protein intake in community-dwelling older people, which fulfills the needs of this specific target group.

Best et al. (14) analyzed the reasons for a low consumption of high-protein foods in older people and identified dental disabilities, reduction in chemosensory and physical abilities, and changes in the living situation, such as becoming a widow, as reasons for the low consumption. The ability to chew and swallow products such as meat and nuts became difficult, and individuals lacked the desire to purchase and cook food for just one person (14). Recent literature indicated that older people (>65) are only partially interested in cooking: only 72% enjoy cooking (15). Nevertheless, another study from Germany identified that 98% of independently living older people (>65) regularly eat three meals per day (16). Tieland et al. (17) examined the dietary protein intake of Dutch older people (average age, 78.6 years) and concluded that beverages were among the top five dietary protein sources of snacks but only contributed to 20% of the daily protein intake (17).

van der Zanden et al. (18, 19) found that—despite a general skepticism toward potential protein-enriched functional foods (18, 19)— dairy products were one of the best alternatives for protein-enriched foods for consumers aged 55+ (18). However, in another study, this research group showed that older people preferred to get their protein via traditional meals (19). van der Zanden et al. (18) recommend developing a tasty and healthy protein-enriched food in order to support the health status of older people (18).

Thus, we concluded that the daily consumption of ready-to-consume protein drinks could be an option to increase protein intake in older people and therefore support independent living. Since the drinks must neither be cooked nor chewed, including dairy protein and as a beverage are meal part for regular meal situations. Such a protein drink does not yet exist on the German food market. The existing protein-rich drinks that meet the recommended minimum amount of 8 g protein per 100 ml (20) are either offered in the form of medical nutrition products for malnourished patients [oral nutritional supplements (ONS)] or advertised as nutritional supplements for athletes.

Additionally, new or improved nutritional products (21) have partially flop rates of 30–90% (22). These high flop rates can be attributed to unmet needs of the target group; consumers should be involved in product development or modification (22, 23).

We were especially interested in autonomous relatively fit community-dwelling older persons, whose decisions were still self-determined in order to be able to preventively influence the health status of the target group. Since protein drinks are rich in protein, they could be a means of increasing protein intake and thus preventing sarcopenia and support independent living of this target group. Literature indicates that a liquid drink for the older population might be an adequate product type (14, 17), as older people can have problems chewing and furthermore do not like cooking regularly, for reasons such as being widowed. The drink should not require any cooking and should include dairy protein and be a beverage and therefore a traditional meal-part for regular meal situations.

Thus, the aim of this study was to gain insight into the aspects, which influence the perception of commercial protein drinks with at least 8 g of protein per 100 ml from community-dwelling older people to understand their needs and find an adequate method for appropriate protein intake at this stage. For this purpose, we asked the participants of the focus groups about general aspects of buying and consuming healthy foods and the importance of such foods for one's own diet in order to better understand how a health-promoting product, such as the protein drink, should be designed to meet the perceived criteria of a healthy food of our target group.

The results of the analysis can be used to develop a protein-rich product, which is suitable for community-dwelling older people, who can—with the help of that product—live independently as long as possible.

In this study, we understand the term *perception* as a process of information processing, through which absorbed environmental stimuli (information intake) is decoded and interpreted. In combination with other information, the processing ends up in subjective internal pictures (24).

## Materials and Methods

### Procedure

In order to pursue our target, three focus groups (*n* = 25) were performed using a semistructured interview guideline; requests by the interviewer were always possible. In all focus groups, participants sat around a table in a separate room with the facilitator, who guided the focus groups; an assistant, who supported the facilitator with the organization during the focus groups; and the secretary, who recorded the focus groups. The facilitator was not especially trained for the focus groups but is experienced in moderating different types of group discussions. The focus groups lasted between 2 and 2.5 h and were conducted without interruption. The focus groups were audiotaped, whereby all of the participants gave informed consent.

At the beginning of each focus group, participants received a short introduction about the protein consumption needs of older people. Afterwards, participants introduced themselves and answered a brief warm-up question about what is important to them in regard to their nutrition. Due to organizational reasons and the experience from the first focus group, the interview guideline was slightly modified after the first session to better fulfill the expectations of the focus groups.

The first focus group (*n* = 7) continued with a 5- to 10-min product handling phase of the protein drinks. During the product handling phase, three different conventional protein drinks (sport drinks and ONS) were tested in all three focus groups. First, each participant received ~100 ml of each drink with refills available. The protein drinks were handed out in plastic cups, so participants could see the drinks but could not identify them. During the product handling phase, participants were requested not to talk about their impressions of the drinks with other participants. Product handling was followed by a discussion of the advantages, disadvantages, and potential wishes for the drinks. Afterward, participants had to fill in a questionnaire about the hedonic perception of the drinks. Then, participants had to name reasons why it was meaningful or not meaningful to consume the drinks. The focus group was completed with a 1-min statement by each participant, concluding their most important considerations with regard to the protein drinks.

After the introduction, the second and third focus group (*n* = 18) proceeded with a discussion about the main influencing factors when choosing healthy groceries. Participants were asked to individually write down all relevant influencing factors on cards. Subsequently, the answers were pinned on a flipchart and discussed with the group. In the follow-up question, the general understanding of the term “health” was discussed. These questions were asked to understand the nutritional context of the participants, their behavior, interest, and thinking in relation to nutrition and health and to link this information to the perception of commercial protein drinks by the older people in the focus groups.

Then, the second and third focus group continued with the product handling phase, in which participants were asked to answer the hedonic questionnaires (see above). This was followed by a discussion of the sensory advantages and disadvantages of the three tested protein drinks and the general necessity and adequacy of such a drink from the participants' point of view. Additionally, participants were asked to describe how their ideal protein drinks should be. They ended with presenting their 1-min statement.

Finally, all except one participant (*n* = 24) filled in a questionnaire about sociodemographic characteristics and their subjective health.

Since the participants of the third focus group reiterated the opinions, explanations, and arguments stated in focus group 1 and 2, saturation was reached. Thus, we did not conduct a fourth focus group.

### Participants

In total, 25 persons aged 75+ participated in the focus groups, consisting of seven to nine participants each. We recruited independently living older people with similar living conditions from three different locations.

The first focus group was carried out in an assisted living home for older people in the City of Straubing, where the participants live independently, having full charge of grocery shopping. Participants were recruited by the staff of the assisted living home and the first author. The second focus group was conducted with independently living people in Nuremberg at the Institute for Biomedicine of Aging, where participants were contacted by the second author based on an address list of participants from previous studies. The third focus group was held in a gym in the City of Straubing with independently living older people, recruited by the staff of the gym.

For all three focus groups, the only criterion for inclusion was an age of at least 75 years. The reason for this age limit is the fact that people at high risk (75+) are generally more likely to experience a negative trend, so the need for prevention is the most pressing in this age group. However, three people were allowed to participate, although they did not meet this criterion (age 67, 68, 74).

### Materials

#### Questionnaires

In the hedonic questionnaire, participants had to answer questions about the overall opinion of the taste, the aroma, the characterization of the aroma (e.g., salty, soft, fruity…), the degree of sweetness, the intensity of the flavor, the texture, and the characterization of the texture (e.g., highly fluid, viscous…) for each drink. Participants were also asked to indicate an overall score for each drink, to state how much of the particular drink they would consume per day and how much they would be willing to pay for each drink. The questionnaire was in the form of a 5-point Likert scale with the answer options “very…,” “quite…,” “mediocre…,” “not…,” and “not at all….” With regard to the characterization of the texture, participants were asked to tick one answer out of seven possibilities, which were thin, viscous, semifluid, creamy, sticky, slimy, and greasy. When asked about the characterization of the aroma, participants were allowed to tick more than one of the 14 offered options. Thus, the latter question meets the criteria of check-all-that-apply (CATA) questions. Per to Ares et al. (25) it is important to consider some rules for these kinds of questions. Hence, we decided not to structure the answer options in alphabetical order as well as to use separate questions with few terms. We also instructed the participants to finish product handling first and then to answer the questionnaire in order to get an overall impression of the different drinks (25, 26).

The sociodemographic questionnaire contained questions about sociodemographic data, the subjective importance of healthy nutrition, the interest in information about nutrition, as well as questions about the personal estimation of each participants' self-description of their objective health absolutely and in comparison, to other older people. For the health-related questions, a 4- or 3-point Likert-like scale was applied. One participant did not answer the sociodemographic questionnaire.

#### Protein Drinks

We decided to test only three protein drinks, since the duration of the focus groups, especially for the age of the target group, already took a long time and we did not want to overstress the participants and thus compromise the validity of our results.

For product handling, participants received three different conventional protein drinks, which were purchased on the Internet or in grocery stores. For the choice of the tested commercially available protein drinks, three selection criteria were applied. First, the protein drinks had to have at least the recommended minimum amount of 8 g protein per 100 ml. Second, they should derive from a similar flavor. It was decided to hand out drinks with fruit flavor, since fruits are associated with health. The third selection criteria referred to the type of products, which had to comply with different product types from the main classes sport drinks and ONS, as these two types comply with the nutritional demands and are commercially available; to reveal the potential differences in perception. Furthermore, both categories were low volume and contained a high amount of protein. Further details concerning the nutrient content of the tested protein drinks are given in Table 1.

**Table 1:** Detailed information about tested protein drinks.

**Nutrition facts**	**Protein Drink A per 100 ml**	**Protein Drink B per 100 ml**	**Protein Drink C per 100 ml**
Type of drink	Sport drink	ONS	Sport drink
Flavor	Raspberry and Blueberry	Multifruit	Strawberry
Protein type	Whey protein concentrate or isolate	Milk protein	Skimmed milk concentrate
Energy	231 kJ/55 kcal	630 kJ/150 kcal	255 kJ/61 kcal
Fat	0.7 g	6.7 g	0.1 g
Saturated fat	0.5 g	0.6 g	0.1 g
Carbohydrates	4.1 g	12.4 g	4.9 g
Sugars	3.5 g	7.1 g	4.8 g
Protein Content	8.0 g	10.0 g	10.0 g

Two of the protein drinks were sport drinks (flavor: raspberry and blueberry = drink A; strawberry = drink C), while the third was an ONS (flavor: multifruit = drink B) fortified with omega 3 fatty acids.

### Data Analysis

The focus groups were audiotaped and transcribed verbatim. Qualitative content analysis in the form of a summary, as described by Philipp (27) was applied by the first author to analyze the data (27). The analysis is based on those parts of the focus groups, which dealt with the general health issues and the product handling phase with its subsequent discussion. According to Mayring (27) and in order to reveal the central themes of the qualitatively collected data and to summarize them, there are three steps to go through, namely, paraphrasing, generalization, and reduction; the latter can be run through several times (27). In our study, we went through the reduction phase twice in order to filter out the actual consensus on the main issues and reduce it to the essentials. By applying this procedure, one moves away from the actual statements of each individual, and the consensus in the group discussions was summarized. The results of this procedure are described in *Results* and reveal, among other things, essential criteria that are important for the target group when buying healthy food. These aspects are linked to the protein drinks, as these are also supposed to be a health-promoting food. Hedonic and sociodemographic data were analyzed using IBM SPSS Statistics 23.

## Results

We will present the results of the focus groups in a logical structure. Thus, they are not ordered in the same way as the questions were asked during the focus groups. We will use quotations to demonstrate and underpin our results and interpretations. It can be noted, however, that the answers of the participants were generally fairly consistent among the three focus groups.

### Characterization of the Participants

In total, 14 women and 11 men (age, 78.6 years ± 4.9; body mass index (BMI), 26.6 ± 4.2) participated in the focus groups (see Table 2). One participant did not answer the socioeconomic questionnaire.

**Table 2:** Characterization of the participants I.

**Topic**	***N***	**Categories**	
**Highest degree of educational qualification**		**Vocational training**	**University degree**
Female	14	7	4
Male	10	7	3
Total	24^*^	14	7
**Household size**		**One-person household**	**Two-person household**
Female	14	9	5
Male	10	3	7
Total	24^*^	12	12
**BMI**		**Minimum**	**Maximum**
Female (average: 25.8)	13	18.3	31.2
Male (average: 27.7)	10	22.2	37.1
Total	23^**^		

*Source: own data set and calculations*.

* *One participant did not answer the socioeconomic questionnaire*.

** *Two missing answers*.

The highest degree of educational qualification of most of the participants was either vocational training or a university degree: 14 persons had some kind of vocational training (vocational training or master craftsmen), whereas seven persons had different kinds of university degrees (polytechnic degree, university degree or doctoral degree). Three persons indicated to have another educational qualification.

There were two different types of living situations present in the focus groups, either 12 persons who lived alone (mostly because they were widowed) or those 12 participants who lived with their partners.

According to the World Health Organization (WHO), there are three different weight statuses adults can have: adults of normal weight (≥18.5 kg/m^2^), preobese adults (≥25 kg/m), and adults who are obese (≥30 kg/m^2^) (28). As shown in Table 2, the participants of our focus groups had a BMI between 18.3 and 37.1 kg/m^2^, with eight persons being people of normal weight, nine people were preobese, and six persons were obese.

As shown in Table 3, 23 participants rated their subjective health as “good” or “very good” and only one person answered that question with “bad.”

**Table 3:** Characterization of the participants II.

**Topic**	***N***	**Categories**			
**Subjective health**		**Bad**	**Good**		**Very good**
Female	14	0	13		1
Male	10	1	8		1
Total	24^*^	1	21		2
**Importance of a balanced nutrition for well-being in old age**		**Less important**	**Important**		**Very important**
Female	14	0	5		9
Male	10	1	4		5
Total	24^*^	1	9		14
**Interest in nutritional information**		**Not at all**	**Not**	**Strong**	**Very strong**
Female	14	0	1	8	5
Male	10	1	2	7	0
Total	24^*^	1	3	15	5

*Source: own data set and calculations*.

* *One participant did not answer the socioeconomic questionnaire*.

In addition, 23 participants found that a balanced nutrition is “very important” or “important” for well-being in old age. One found nutrition a “less important” factor for well-being.

Finally, five participants of the focus groups were very strongly interested in nutritional information, 15 participants showed strong interest in such information, three participants answered that they were not interested, and one participant showed no interest at all in information on nutrition.

### Perception of the Protein Drinks

In the following, the aspects that influenced the perception of the protein drinks during the focus groups will be presented.

#### Distrust Toward the Modern Food Industry and Dietary Recommendations

The first aspect we found that influenced the perception of the protein drinks was that most of the participants were considerably skeptical about the food industry and new types of food products in general. Apparently, this issue was highly sensitive, as this comment emphasizes:

“We live in a society of profit and everyone tries to bring as much as possible on the market.”

Not only skepticism but also confusion and uncertainty about dietary recommendations played an important role for the distrust in the modern food industry.

One person stated:

“Sometimes coffee is harmful, sometimes it is not.”

Then another person said:

“You should not eat too much butter. Others say you should eat it.”

These statements pointed out that certain skepticism about the modern food industry and its products persists within this age group. In addition, the statements illustrated the uncertainty and confusion the participants encountered with regard to current dietary recommendations. Together, these aspects have negatively influenced the perception of the commercial protein drinks, given that they were perceived as being rather modern and coming hand in hand with a dietary recommendation.

#### Important Aspects When Buying (Healthy) Foods

We asked participants (*n* = 18) what was important to them when choosing healthy foods. The participants attached high importance to four aspects: groceries must be natural, the ingredients must be grown locally, vegetables and fruits must be fresh and the manufacturer must be trustworthy. These aspects were not only important for healthy foods, but generally when choosing food products. For example, one older person stated:

“When I see teenagers and I see their shopping baskets, what they buy is a horror to me: ready roasted fried potatoes and, and, and.”

This statement of an old person highlighted the importance of the naturalness and freshness aspects. Even in the first focus group, where we did not explicitly ask these questions, we learned about the importance of natural ingredients:

B1: “So natural raw materials would be more important to me.”

Interviewer: “Yes.”

B2: “Yeah, let's agree to eat something healthy, right?”

B3: “Yes.” (Several agree)

With regard to the origin of the food product, one subject said:

“Well, if I have a choice, from the region.”

An additional important role was the factor of trust, as we already realized when participants talked about the food industry. The following example pointed out this aspect especially with regard to its own nutrition.

“We have a rural butcher, whom I trust completely.”

These outlined statements illustrated that for older people, aspects such as naturalness, freshness, locally grown ingredients, and trust played an important role when buying (healthy) foods, and they were of prominent relevance for the general perception of food products.

#### General Lack of Knowledge Concerning Protein and Special Protein Requirements

Another result we found that influenced the perception of the commercial protein drinks was that nearly all of the participants lacked a general knowledge about protein and its function in their body, as the following example illustrated:

“And when he says you lack…” Interviewer: “…protein.” Older person: “…what is there inside?”

Additionally, the persons who showed a lack of knowledge about protein also did not know about the daily protein requirements in old age. A person asked for example:

“May I ask what the health benefit of this product should be?”

In general, the emphasized statements from the different focus groups showed that the older people neither knew for themselves about the physiological function of protein nor about the need for additional protein consumption at their age group despite the probable changes that come with age, such as the loss of muscle mass. This aspect additionally influenced the perception of the commercially available protein drinks, since the participants questioned the need of protein drinks in general.

#### Perceived Lack of Necessity to Supplement Protein

Even afer giving the participants information about their special protein requirement, they did not see the necessity of consuming additional protein. One individual mentioned:

“I think my food is already full enough with proteins. I don't need an additional one.”

One explanation for this opinion might be the subjective health status of the participating persons (see Table 2). In line with that, participants did not see a need for protein supplementation due to their current, healthy lifestyle, and diet. Thus, participants could not identify any benefit a protein drink could offer them. They felt that their current diet and lifestyle was covering all their nutritional needs.

In contrast, there was only one participant who knew sufficiently about the need to consume additional protein.

“But I heard about everything you can do with such proteins [powder] that tastes good. As an older person myself I had to get more information about this.”

The majority of the participants did not see the necessity of supplementary protein due to their subjective health status even after giving them information on that topic, and their request for doctoral diagnosis revealed the skepticism about special protein requirements and the need to additionally consume protein-rich food or drinks.

#### Evaluation of the Protein Drinks

**Figure 1:**
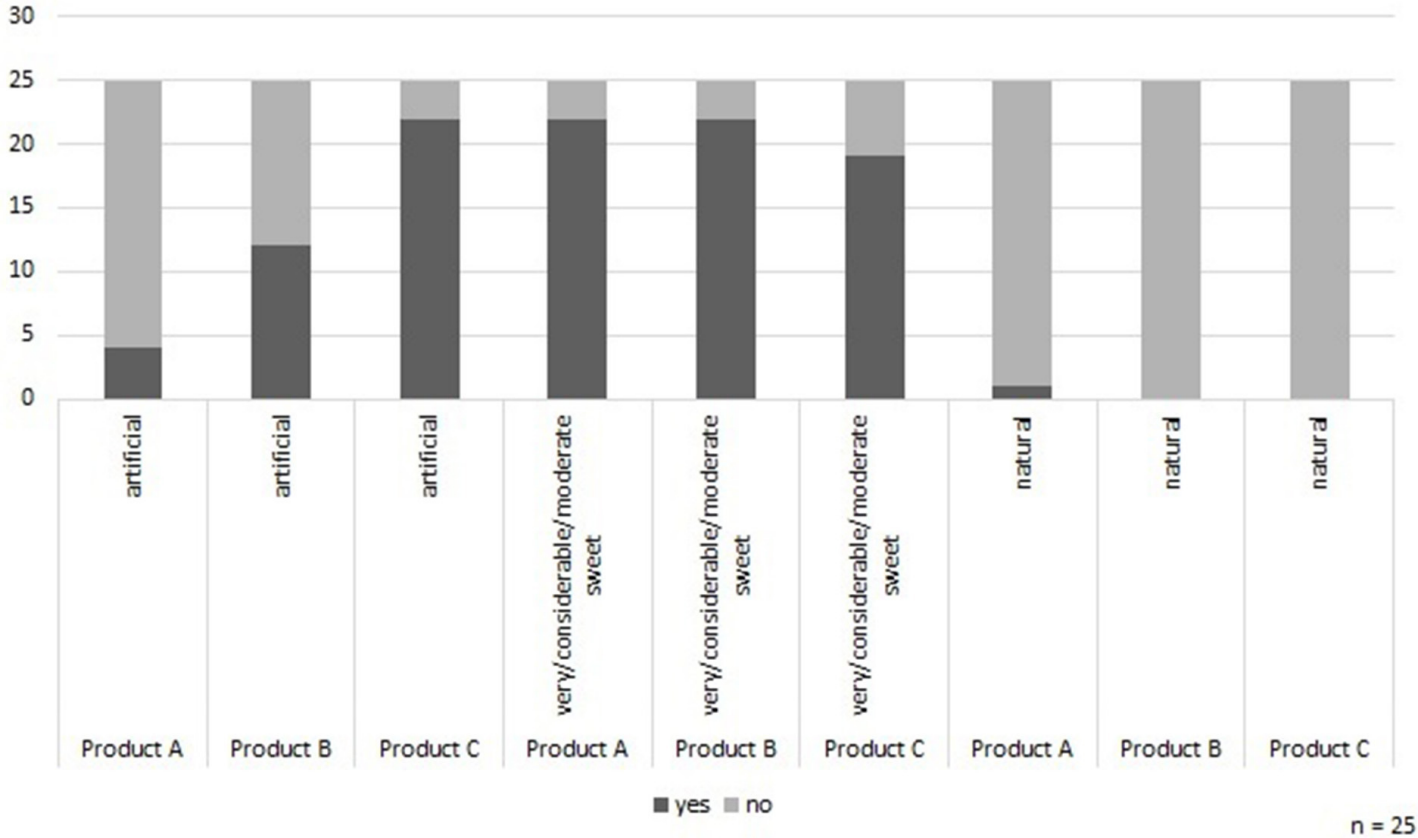
Assessment of particular factors of the protein drinks. Source: own data set and calculations.

As shown in Figure 1, the selected protein drinks were all rated as rather sweet and not natural. Moreover, only Product A was not perceived as artificial, the evaluation of Product B was relatively balanced in this regard, whereby Product C was rated as being artificial by 22 participants.

The following statement showed the importance of natural products for this age group especially with regard to the commercial protein drinks:

“Too spurious, too perfumed, too artificial.”

Another participant stated:

“Well, for me all of them are too sweet, way too sweet.”

Being asked, whether or not the product would be interesting for the target group, one individual pointed out:

“No, because it has to be a pleasure to drink it.”

This quotation illustrated that none of the drinks met the pleasure aspect in the consumers' point of view.

**Figure 2:**
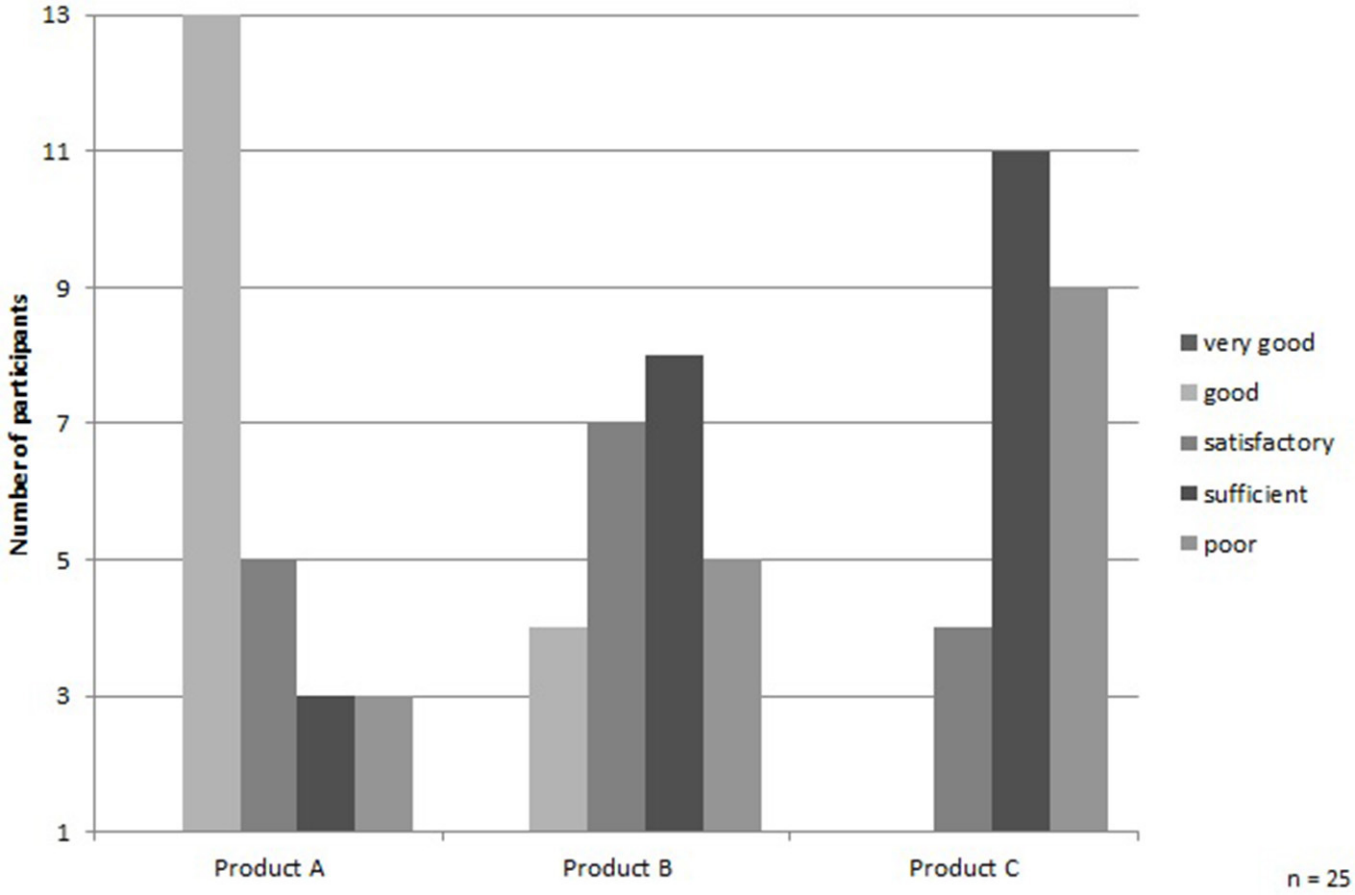
Final evaluation of the protein drinks. Source: own data set and calculations; notes: 1 represents “very good” and 5 represents “poor.” The grade point average of the drinks is Product A = 2.7, Product B = 3.4, and Product C = 4.1.

Altogether, the “favorite” protein drink was the raspberry and blueberry flavored sports drink (Product A) (see Figure 2). The drink was rated as “fresh” and “fruity” and had a sweet-sour component, which most of the participants liked. The medical nutritional supplement with the flavor of multifruit (Product B) is the second most preferred. However, this drink also polarized the most, whereas the other two analyzed drinks were highly strong and clear in perception (Figure 2). None of the participants liked the sport drink with taste of strawberry (Product C): it was rated as too artificial and was far too sweet according to the respondents.

With regard to the texture, the participants agreed that this feature of the protein drinks did not vary substantially across the different products. Protein drink A was perceived by 11 people as thin, by seven as creamy, by four as semifluid, and by the rest other perception of consistencies occurred. Protein drink B received six votes for thin, 14 votes for creamy, and the rest were distributed among other perceptions of consistency. Finally, the participants perceived protein drink C 15 times as thin and 5 times as creamy; the rest of the votes were distributed to other consistencies.

The results of product handling of all three focus groups showed that, with regard to liking the drinks, the identified four important aspects: naturalness, freshness, locally grown ingredients, and trust when buying (healthy) food products mattered for the evaluation of the commercially available protein drinks.

Overall, the tested protein drinks were not pleasurable for the participants to consume. They were too sweet, too unnatural, and too unhealthy. They would only buy or consume the drinks if they meet the described important aspects of buying (healthy) food products, namely, naturalness, freshness, locally grown ingredients, and trust.

#### Protein Drinks Cannot Be Integrated in Daily Nutrition

Another aspect that influenced the perception of the protein drinks was that the participants could barely imagine how to integrate the drinks in their everyday life. Furthermore, participants did not see themselves as potential consumers of these drinks since the drinks did not fit into their lifestyles or daily nutrition plans. The following statements characterized this perception:

“What kind of beverage shall it be […], just drinking it if thirsty or am I supposed to drink it somehow after eating, or before […]?”

Reasons for not knowing when to consume the drinks were first the taste, since the drinks were too sweet and did not correspond to their nutritional habits. Conversely, the unseen necessity to consume additional protein led to unwillingness by the focus group participants to consume the drinks.

## Discussion

In this study, we conducted three focus groups (*n* = 25) to evaluate aspects that influence community-dwelling older people's perception of three commercially available protein drinks with at least 8 g of protein per 100 ml. We were especially interested in autonomous persons, whose decisions were still self-determined in order to find a product that can be readily added to the diet of older people to prevent frailty or other ailments caused by inadequate protein intake. Therefore, it is essential to understand the needs of this target group, who would voluntarily consume the protein drink.

The first perception influencing aspect of the protein drinks we found was a skeptical attitude toward the modern food industry. Second, we found fresh, natural foods, locally grown ingredients, purchased from a trustworthy manufacturer or distributor to be important factors for our target group. The latter explains why the participants were rather skeptical about the modern food industry and its food products, as both often do not comply with the requested criteria from the participants' point of view.

Literature confirmed the finding that groceries should comply with these criteria. Proximity to the manufacturer is an important aspect especially for older people in order to bypass agro-industrial conditions, whereas in contrast, the traditional country life is idealized, since it stands for health and naturalness (29). Furthermore, Steptoe et al. analyzed a correlation between age and the interest in consuming products with natural ingredients and the rejection of additives, whereas Roininen et al. confirmed this fact by finding a connection between older people and the use of natural products (30, 31).

The confusion and uncertainty we found toward dietary recommendations and in particular toward the protein topic could be explained by the partially conflicting food-related information that older people are confronted with and what leads to that confusion. As a result, participants lacked knowledge about protein in general, although 20 participants stated they were very strongly or strongly interested in nutritional information. The literature cannot confirm this lack of knowledge, since most studies dealing with protein intake in older adults did not consider the knowledge aspect. Only van der Zanden et al. (18) dealt with this aspect and concluded that the participants did have knowledge about the importance of protein consumption, but not on its special physiological effect (18).

In our study, even after informing the participants about their special protein requirement, they did not see the necessity of consuming additional protein. The participants perceived their diet as already covering all their needs, which is also reflected in their perceived health status. A similar result was found by other researchers: the subjective health status is a probable barrier to change personal consumer behavior because of a perceived lack of necessity (18, 32). Moreover, if people do not consider themselves to be at risk for a certain threat, they would not be motivated to protect themselves (33), thus deeming the product unnecessary.

As another outcome of our study, the unperceived necessity to consume additional protein is reflected in the stated need for confirmation of increased dietary protein intake by a doctor. Considering the fact that participants were not convinced by the dietary recommendations given during the focus group, yet participants stated interest in doctor recommended dietary guidelines, also found by van der Zanden et al. (18) and Korzen-Bohr and O'Doherty Jensen (34), showed the particular importance of trust for older people. However, consumers' trust in the food industry is lacking (34).

It is unsurprising that people and especially the older generation (aged 75+) feel overstained with conflicting food related information, the wealth of dietary recommendations, and the abundance of food nowadays, since this was not always the case during their life (35). This uncertainty should be considered when intending to support an appropriate amount of protein intake in older people within this food system and with a rather modern product.

Combining these results with the fact that a protein-dense drink can be used preventively and should be consumed when people still live independently, the significance of a suitable communication (strategy) becomes very apparent. We already discussed that people do not see the necessity of changing their behavior if they do not feel the consequences of it. Particularly knowing this aspect, the importance of suitable and continuous communication about the importance of adequate protein consumption for optimal longevity and independent living with a high quality of life becomes even more obvious.

That is why nutritional communication should turn away from a sender–recipient relationship, where communication is one-sided and individuals are understood as rational consumers, who only make knowledge-based decisions (36). Instead, there is an urgent need to implement communication at eye level for older people, which considers their ideas of a credible health elucidation. The specific ideas and wishes of older people in this area should be investigated in future studies. Additionally, they should be asked in such studies whether older people are interested in gaining specific knowledge in protein (enrichment) in their diets and physiological functions and in which way such a knowledge can be transferred.

As a result of our study, one possibility to avoid a sender–recipient relationship, where older people do not have any direct contact person to rely on, could be the collaboration with family doctors. The participants of our focus groups indicated that they would trust the opinion and suggestions of their family doctor and including their dietary recommendations. Since the majority of older people visit their family doctor frequently (37), the collaboration could be one manageable way in an atmosphere that promotes trust to explain the need to consume a certain amount of protein even if the person feels healthy. As van der Zanden et al. (18) found similar results, this approach seems to be promising but is rarely considered in health care (18).

Furthermore, the majority of the participants did not like the protein drinks. If at all, most of them preferred the one that tasted the most fresh and fruity. This also supports the decisive influence of freshness and naturalness when buying food products. Arens-Azevedo and Behr-Völtzer (38) confirmed in their *textbook nursing care*, the reasons for food choice in older people being taste and pleasure, health aspects as well as compatibility, and habit (38). From the consumers' point of view in our study, neither pleasure, health, compatibility, nor habits were fulfilled by the commercial protein drinks. Only in the case of Product A (flavor: raspberry and blueberry) taste has been rated as good by the majority of the participants. However, simply reasonable taste of one of the tested drinks could not motivate the respondents to consume the protein drink frequently in the future. Therefore, after the consideration of the identified important aspects for the protein drink and its development, further studies with different flavors of protein drinks should be conducted with the target group.

One aspect that is continually mentioned in the literature is the heterogeneity in older people due to their long and different life experiences (39–41). With regard to our study, a significant differentiated perception within the target group was not observable. The respondents were all relatively consistent in their knowledge and perception concerning commercial protein drinks, as well as in regard to the important aspects they attached to food. Thus, we assume our results as valid, since consistency and repetition within the groups was shown.

### Methodological Considerations and Limitations

In this study, the method of focus groups was chosen to gain deeper insights into consumers' perception of protein drinks. Since face-to-face interviews were conducted respondents were able to make requests, which add to the holistic understanding of the participants' perceptions. Furthermore, within focus groups the development of group dynamics is possible, so that polarizations arise and the reasons for certain opinions become clear. As we wanted to gain deeper insight into the factors influencing the perception of commercially available protein drinks by older people, the method of focus groups seemed to be highly suitable.

However, a limitation when conducting the three focus group refers to the sequence during product handling. As we first handled the product tasting, followed by a discussion and then the completion of the questionnaires for the respective protein drinks, we cannot rule out a bias due to the joint discussion regarding the answers to the questionnaires for the three protein drinks. The reason for this procedure was the fact that the same danger of bias also exists the other way around. If the questionnaire had been filled out first and discussed afterwards, focusing on the aspects covered by the questionnaire by the participants could not be excluded. However, our aim was to get a basic feeling for the perception of protein drinks and to listen to the participants independently of the hedonic test and let them discuss the aspects most important to them.

This study was performed with a relatively small sample, which is a state-of-the-art approach when working with focus groups. Thus, a generalization of the older population in Germany cannot be made based on the results of this sample, and such a generalization was not intended in this study.

Furthermore, protein drinks could appeal more to frail people, as it is probable that they consider themselves to be part of an at risk group due to their awareness of their own frailty. However, since the protein drinks should be viewed as preventative in nature, individuals who are already frail were not the target group for our study.

In addition, the selection criteria of the protein drinks led to a specific and restricted sample of protein drinks mainly due to practical reasons and time requirements in order to not overstress the participants. Other reasons for the choice of the three selected commercially available protein drinks were the ambition for comparability among the drinks and the assumption that fruit flavors are associated with healthy food products. This focus was due to the target to analyze mainly the perception of product-inherent characteristics of protein drinks for older people as basis for developing a corresponding protein drink prototype. However, we have to acknowledge that the assumption that fruit flavors are associated with healthy food products cannot be underlined by the results of our study, since the older people in our focus groups did not associate the tested protein drinks with health or healthy food products. Therefore, the selection of only fruit tastes should be reconsidered in future studies because it limits the choice and excludes and disregards possible flavors that might have received more approval.

## Conclusions

The aim of this study was to gain insight into the aspects, which influence the perception of commercial protein drinks with at least 8 g of protein per 100 ml from community-dwelling older people to understand their needs and find an adequate method for appropriate protein intake at this stage.

According to the presented results, we only found aspects, which negatively influenced the perception of commercial protein drinks. Reasons for these results were a general distrust in the modern food industry, the identified important aspects when buying (healthy) foods, which did not comply with the tested protein drinks, the perceived lack of necessity to supplement protein intake, participants' dislike of the taste and flavor of the tested protein drinks, and a lack of usability of the drinks in the diet routine from the consumers' point of view.

For these reasons, we suggest that a future protein-dense beverage should be significantly less sweet—maybe offered in different flavors—less artificial, fresher, and consisting of more natural ingredients and ideally its ingredients should be grown locally. The latter could also reduce the distrust toward the modern food industry and its products if this aspect is communicated in a trustworthy way. During product development, different types of flavors should be tested by the target group. This could help overcome the lacking pleasure aspect of the drinks by the participants.

When developing a protein drink, communication about this product must be considered carefully. Aspects such as naturalness, freshness, and locally grown ingredients should be highlighted as well as the importance of protein consumption in old age. Thus, communication strategies must be reconsidered and revised, and the development of new strategies should be based on further research on the specific ideas and wishes for a suitable communication from the older peoples' point of view. As a mid-term effect, the negative perception of the drinks could decrease, and more older people would be willing to integrate a protein drink into their dietary routine. The collaboration with family doctors could be a possible step to start an appropriate communication strategy that also builds on the trust that family doctors have among older people.

Since we did not find a general reluctance toward a liquid product, which does not require cooking, we still find protein drinks to be one possible option to increase protein intake in community-dwelling older people. This could be combined with further strategies, for example, to develop other protein-enriched food products that can be integrated into the target group's meals and the technical and organizational routines of the community-dwelling homes.

## Data Availability Statement

The datasets generated for this study are available on request to the corresponding author.

## Ethics Statement

The data protection officer of Hochschule Weihenstephan – Triesdorf, University of Applied Sciences (HSWT) approved the ethics and consent procedures for the study.

## Author Contributions

LL conceived and designed the experiments, analyzed the data, and was the first author and wrote the paper. LL and KM performed the experiments. AH, EK, and DV provided information on the nutritional background of the project, selected the test drinks, contributed to the development of the questionnaires, and drafted and revised the manuscript. All authors contributed to the article and approved the submitted version.

## Conflict of Interest

The authors declare that the research was conducted in the absence of any commercial or financial relationships that could be construed as a potential conflict of interest.
